# Refractory neovascular age-related macular degeneration: time-dependent changes of central retinal thickness with anti-VEGF treatment

**DOI:** 10.1007/s00417-020-05000-3

**Published:** 2020-11-27

**Authors:** Marta Zola, Elisa D’Alessandro, Mohamed Sherif, Audrey Nguyen, Dominique De Azevedo, Céline Haeller, Edwige Forestier, Irmela Mantel

**Affiliations:** Department of Ophthalmology, University of Lausanne, Jules-Gonin Eye Hospital, Foundation Asile des Aveugles, Lausanne, Switzerland

**Keywords:** Aflibercept, Anti-VEGF, Central retinal thickness, Neovascular age-related macular degeneration, Optical coherence tomography, Ranibizumab

## Abstract

**Purpose:**

To assess the influence of time interval since last injection and time from baseline on central retinal thickness (CRT) in neovascular age-related macular degeneration (nAMD) with fluid refractory to monthly anti-VEGF treatment.

**Methods:**

This retrospective study included nAMD eyes with incomplete response to anti-VEGF defined by the presence of intra- or subretinal fluid on optical coherence tomography despite maximal (monthly) anti-VEGF dosing. The outcome measure was CRT, and two time variables (time from last injection ant time from baseline) were the independent factors included in the individual correlation analyses. In addition, an association analysis was performed.

**Results:**

Sixty eyes of 56 patients (67.9% females, mean age: 78.7 ± 6.8 years) were included with a mean included time period of 35.6 months. A significant positive correlation between CRT and the time from last injection occurred in 24 (40%) and 25 (42%) eyes by univariate and multivariate analysis, respectively. Time from baseline was significantly correlated with CRT in 29 (48.3%) and 30 (50%) eyes by univariate and multivariate analysis, respectively. This correlation was positive in 12 (20%) and negative in 18 eyes (30%). No association with such correlation was found.

**Conclusion:**

So-called refractory nAMD frequently shows a correlation of CRT with the interval in days from the preceding anti-VEGF injection, revealing that there is a subgroup of short-term responsiveness of the residual fluid. Moreover, slower CRT changes may occur over the years, either decrease or increase. In case of a slow CRT increase, this might require a diagnostic workup and therapeutic change.

**Supplementary Information:**

The online version contains supplementary material available at 10.1007/s00417-020-05000-3.

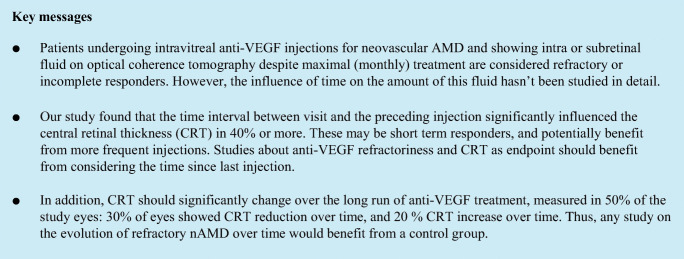

## Introduction

Age-related macular degeneration (AMD) is a chronic degenerative macular disease and a major cause of visual loss in the elderly population in the industrialized countries [1]. The current standard of care consists of intravitreal injections of anti-vascular endothelial growth factor (anti-VEGF) [2, 3]. Different guidelines and consensus documents establish the importance of using both anatomical and functional parameters to guide the management of this disease [4, 5]. Optical coherence tomography (OCT) has become the single most important examination for determining the need for retreatment, based on the presence or absence of sub- or intraretinal fluid—a sign of CNV activity. The response to anti-VEGF treatment varies widely, as does the need for retreatment [6]. It has been shown in various clinical studies that intra- and subretinal fluids persist to a certain degree in some eyes with nAMD under treatment with anti-VEGF drugs [7]. Eyes with fluid at monthly visits despite monthly injections have been referred to as refractory or incomplete responders. Due to their incomplete response, they have been subject to anti-VEGF agent switching or adjuvant treatments [7, 8]. The corresponding reports usually compared anatomical outcomes before and after treatment change, without control groups. Thus, time parameters may act as potential confounders. These include the precise time interval from preceding injection and the time passing by from injection to injection, which can be expressed as time from baseline. However, it is not known to what degree these parameters have an influence in these cases on the commonly used outcome parameter central retinal thickness (CRT).

In order to better understand incomplete responders, it appears important to understand their time-dependent behavior. We hypothesized that in a subgroup of patients, CRT may depend on the number of days from preceding injection. These cases may show their maximal response earlier than on the usual monthly monitoring visit, and they may be responders at earlier time points. In addition, we hypothesized that the treatment response may evolve over multiple injections. For these reasons, this study aimed to investigate the influence of time on CRT measurements, both in terms of interval from the preceding injection and as time from treatment initiation, in eyes with incomplete response despite monthly anti-VEGF treatment.

## Methods

### Study setting

This study was performed as a retrospective chart and optical coherence tomography (OCT) imaging review, in the medical retina department of the University Eye Hospital Jules-Gonin, Lausanne, Switzerland. The study was approved by the Swiss Federal Department of Health for retrospective data analysis and was performed in accordance with the ethical standards of the Declaration of Helsinki.

### Subject and material selection

A consecutive series of eyes with nAMD which showed a refractory time period of at least 12 months during the intravitreal anti-VEGF treatment course was identified. Refractoriness was defined as an incomplete resolution of fluid under maximal monthly treatment with anti-VEGF (aflibercept or ranibizumab), which did not allow for longer treatment intervals to be planned. More specifically, if an eye showed repeatedly the presence of intra- or subretinal fluid on OCT 1 month after the anti-VEGF injection, it was considered to be an incomplete treatment response. These eyes may or may not have complete resolution in between monthly injections, but this was not a criterion for inclusion to the study. Fluid under the pigment epithelium was not a criterion. In addition, small amounts of fluid overlying large fibrotic or atrophic changes were not considered, as these changes were clinically considered to be of degenerative character.

For each candidate eye, the longest period of refractoriness (incomplete response) was identified. This is, between month 3 and the last available follow-up date, we identified the time period during which monthly treatment was indicated due to fluid signs as defined above. Addition requirements were as follows: (1) use of the same anti-VEGF drug, (2) use of the same spectral domain (SD)-OCT machine (Heidelberg Spectralis OCT, macular cube 6 mm, 49 lines, Heidelberg Engineering, Heidelberg, Germany; or Cirrus OCT, macular cube 512 × 126; Carl Zeiss Meditec, Inc., Oberkochen, Germany) during the entire time period, (3) availability of at least 10 consecutive OCT scans during the refractory time period, and (4) refractory fluid located within the central retinal area of the ETDRS grid, and thus measurable as CRT. If an anti-VEGF agent switch was performed, the refractory time period for only one drug was included, opting for the longer period with fulfilled inclusion criteria. Exclusion criteria were (1) insufficient SD-OCT images for CRT determination, (2) any adjuvant treatment during the refractory period and the year before, (3) any intraocular surgery during or 6 months before the refractory period, and (4) any confounding retinopathy.

### Study parameters

All available OCT scans from the refractory period of each included patient were analyzed. First, they were reviewed for segmentation and manually corrected (IM, AN, CH) if needed using the inbuilt segmentation tool: all b-scans traveling through the central retinal area of the ETDRS grid (plus 2 b-scans above and below) were reviewed and corrected for the internal limiting membrane and the retinal pigment epithelium layer (Cirrus OCT) and Bruch membrane (Spectralis OCT), respectively. Thereafter, the corrected CRT measurements were recorded, along with the date of the acquisition, the OCT machine used, and the date and drug type of the immediately preceding intravitreal injection in the same eye, with its number within the treatment series. Thus, a series of consecutive data was obtained for each eye, enabling individual statistical analysis for both outcome measures: the correlation of CRT with the time interval from last preceding injection in order to determine the cases with early responsiveness, and the correlation of CRT with time for treatment start, in order to determine the frequency of slow progressive changes, potentially related to drug tolerance of changes in VEGF secretion. Additional data collected were date of birth, sex, and date of first treatment with anti-VEGF in the study eye. Furthermore, we collected information about the type of fluid considered refractory: intraretinal fluid or subretinal fluid or both.

### Treatment strategy

The routinely used retreatment strategy was an interval based regimen named “observe-and-plan,” as described previously [9]. The regimen’s rationale is similar to the “treat-and-extend” regimen, where treatment intervals are extended by half a month in the absence of fluid and shortened if the inverse it true. The minimal interval between injections is 1 month, and the maximal planned interval is 3 months. The key difference to the “treat-and-extend” regimen is the application of short treatment plans, including several injections (usually 3) without intermediate monitoring visits.

However, as the data was collected from a real-life intervention cohort, the adherence to the treatment plan was not perfect. Thus, even if the treating ophthalmologist decided within the “observe-and-plan” for monthly retreatment due to incomplete response as described above, patients occasionally postponed their appointment. Such an event was accepted into the study, as long as it was a single event per patient within the study period and that the interval from preceding injection was not above 49 days. Otherwise, the study time period was shortened accordingly, or the patient’s eye was excluded from the study.

Until the end of 2012, ranibizumab only was commercially available. When aflibercept became available at the end of 2012, many refractory patients were switched to the new anti-VEGF agent. Thus, included patients in the aflibercept group had mostly undergone an anti-VEGF agent switch before.

### Statistical analyses

Statistical analysis was performed on each individualized data set. Besides descriptive statistics, a Pearson correlation analysis was performed with CRT as the outcome measure. The time interval from the preceding injection and time from the baseline date (first anti-VEGF injection) were the factors in the correlation analysis. In cases with *p* ≤ 0.2 on both correlation analyses, a multivariate analysis was performed with both the time parameters. A Microsoft Excel 2010 spreadsheet and JMP software for Windows (version 8.0.1, SAS institute Inc., Cary, NC) were used. A two-tailed probability of 0.05 or less was considered statistically significant.

## Results

This study included 60 eyes of 56 patients (67.9% females, mean age 78.7 ± 6.8 years). During the study period, aflibercept was used in 40 eyes (66.7%), and ranibizumab in 20 eyes (33.3%). The OCT machine used during the included refractory period was the Heidelberg Spectralis in 53 eyes (88.3%) and Zeiss Cirrus in 7 eyes (11.7%). The period included for analysis was a mean of 38.5 months (range: 17–53), including a mean of 15.6 (SD 4.2) OCT examinations (Table 1). The residual fluid defining incomplete treatment response included intraretinal fluid in 23 eyes (38%) and subretinal fluid in 42 eyes (70%), both were present in 5 eyes (8%).

**Table 1:** Demographic and clinical characteristics of the included patients and eyes, with respect to the studied anti-VEGF refractory time period in neovascular age-related macular degeneration

Demographics Age (years) Sex, female *n* (%) Laterality, *n* (%) Right Left	78.8 ± 6.8 40 (72.7%) 33 (55%) 27 (45%)
Anti-VEGF drug Aflibercept, *n* (%) Ranibizumab, *n* (%)	40 (66.7%) 20 (33.3%)
OCT Instrument Spectralis HRA + OCT, *n* (%) Cirrus HD-OCT, *n* (%)	53 (88.3%) 7 (11.7%)
OCT measures for analysis Mean number per patient Median (IQR)	15.6 ± 4.2 15 (12–18)
OCT central retinal thickness Mean value over all patients in micrometers	315.2 ± 106.2
OCT fluid type (*n* eyes/percentage) Presence of refractory intraretinal fluid Presence of refractory subretinal fluid	23 (38%) 42 (70%)

VEGF, vascular endothelial growth factor; OCT, optical coherence tomography; IQR, interquartile range

**Fig. 1:**
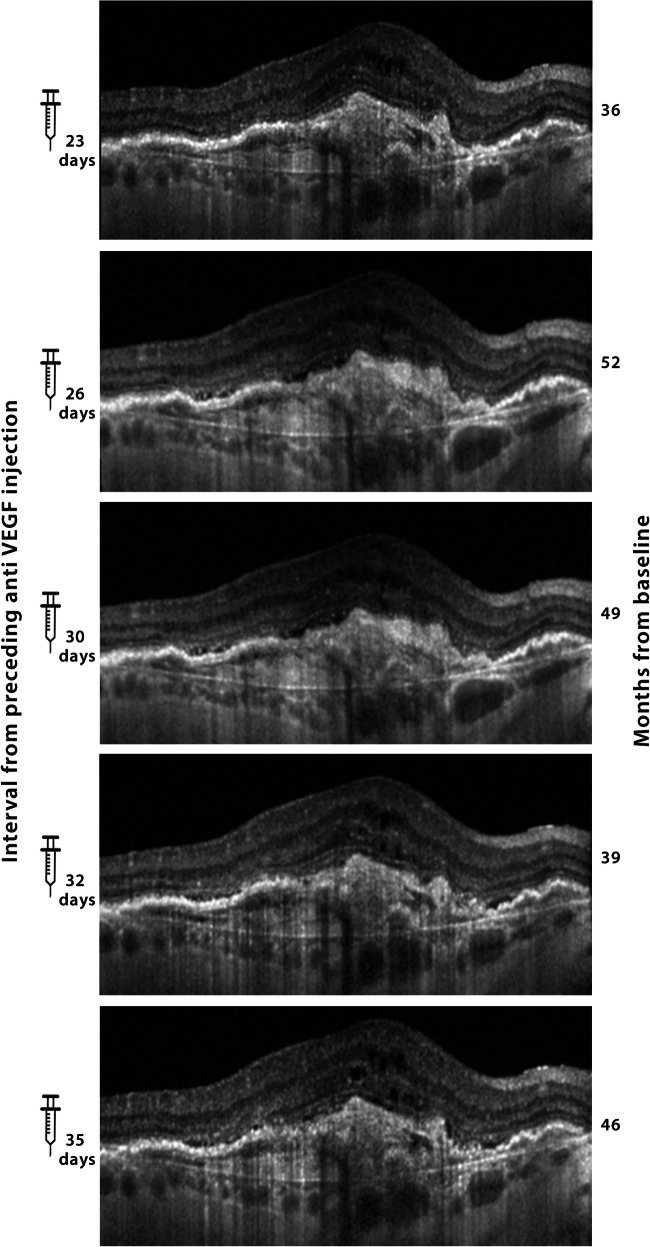
Selected SD-OCT scans of patient 18, showing fluctuations of fluid within a few days of difference. The eye was treated with monthly aflibercept, and the SD-OCT images were acquired on the Heidelberg Spectralis machine. Days from the preceding aflibercept injection are shown on the left, whereas numbers on the right indicate the months from treatment initiation. Small but measurable differences in central retinal thickness (CRT) were seen (CRT of 476, 481, 505, 525, and 514 μm measured after 23, 26, 30, 32 and 35 days from preceding injection, respectively). The analysis of all 10 available study OCT measurements showed a significant correlation of CRT with the time interval from the preceding anti-VEGF injection (r2 0.76, *p* = 0.01)

**Fig. 2:**
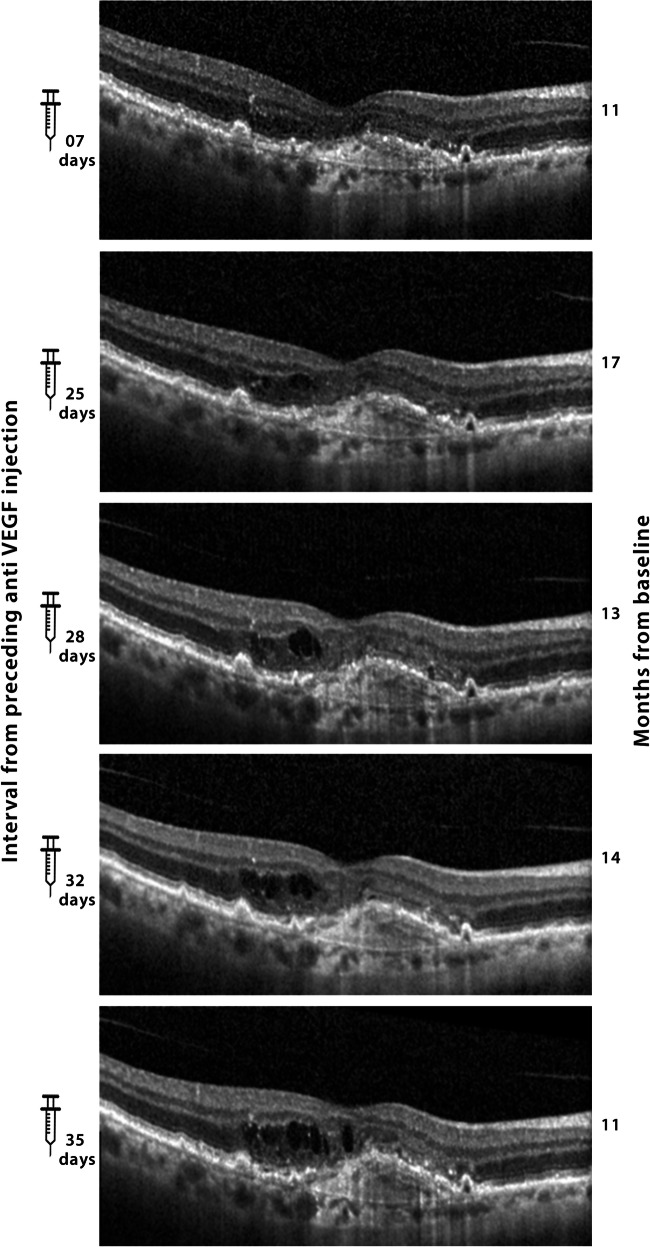
Selected SD-OCT scans of patient 57, showing a transient response to anti-VEGF treatment 7 days after the injection, with early recurrence of intraretinal fluid as seen at monthly visits after anti-VEGF injections (in this eye aflibercept): fluid amounts and therefore also central retinal thickness (CRT) increased dependent on the time from the preceding aflibercept injection. The numbers on the left indicate the time in days from the preceding aflibercept injection. The CRT measured by the Spectralis device was 246, 292, 305, 331, and 355 μm from top to bottom, at 7, 25, 28, 32 and 35 days from preceding injection, respectively. The analysis of all 25 available study OCT measurements showed a significant correlation of CRT with the time interval from the preceding anti-VEGF injection (*r*^2^ 0.46, *p* = 0.01). In addition, a significant correlation with time from baseline was found (*r*^2^–0.62, *p* = 0.001), indicating slow improvement during refractoriness. The latter was confirmed in bivariate analysis

**Fig. 3:**
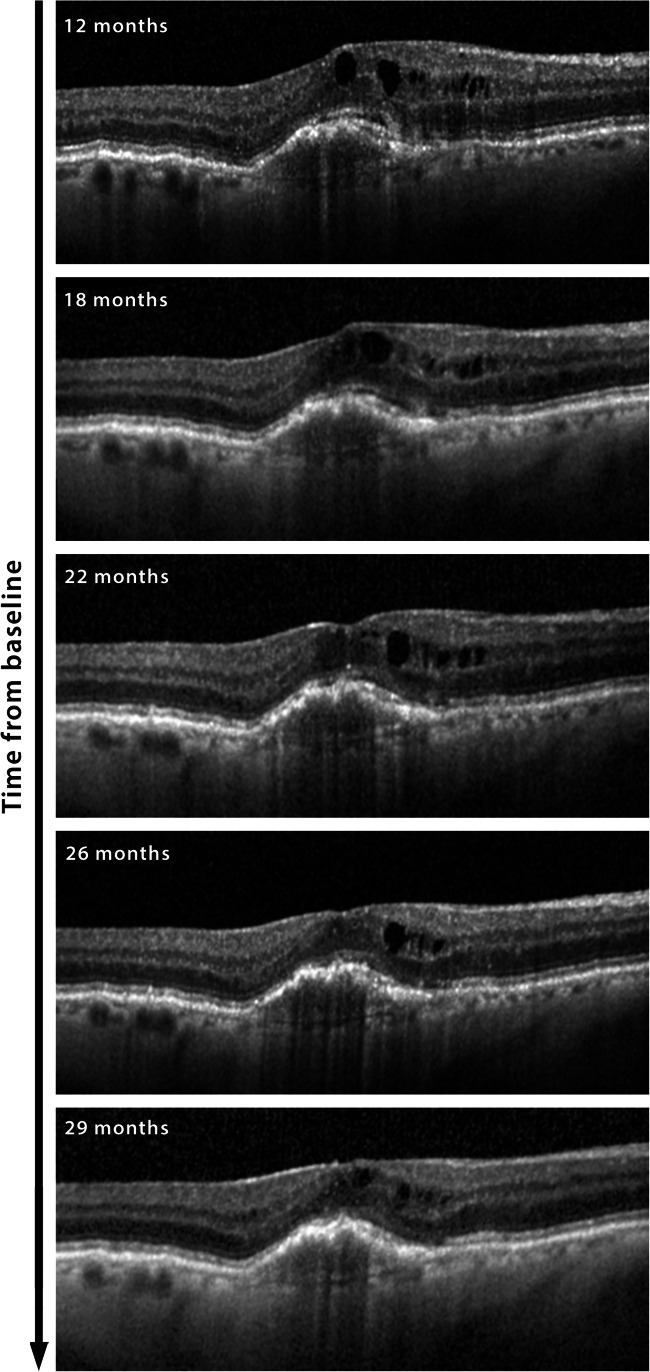
Transfoveal SD-OCT b-scans of patient 2 at selected timepoints of the anti-VEGF refractory study period, showing a slow reduction of the amount of the intra-retinal fluid with time. The numbers on the right give the timepoints in months from treatment initiation. During the illustrated treatment period that patient underwent continuous monthly retreatment with aflibercept, and SD-OCT images were acquired with the Heidelberg Spectralis machine. The automated measurements of the central retinal thickness (CRT) were 329, 312, 369, 293, and 258 μm at 12, 18, 22, 26 and 29 months, respectively. The precise delay from the previous aflibercept injection was 23, 26, 30, 32, and 35 days, respectively. The correlation analysis including all 10 SD-OCT scans available according to the study criteria showed a significant association between CRT and the time from baseline with an *r*^2^ of − 0.93 (*p* = 0.0001)

The individual parameters and outcomes are given in Online Resource 1. An overview of the results and outcome groups are given in Table 2. To summarize, the results showed a significantly positive correlation between the CRT measures and the time interval from the preceding injection in 24 (40%) eyes by univariate analysis. This increased to 25 (42%) eyes after multivariate analysis. Examples of such positive correlation are given in Figs. 1 and 2. These cases may not be truly refractory but rather short-term responders with early recurrence. Thus, they might potentially benefit from a visit after 1–2 weeks in order to be identified. Furthermore, a significant correlation between CRT and the time point from baseline was found in 29 (48.3%) eyes by univariate analysis, which increased to 30 (50.0%) eyes after multivariate results. This correlation was positive in 12 eyes (20.0%), which is an increase of CRT over time as treatment was ongoing, evoking the possibility of drug tolerance. On the other hand, a negative correlation was seen in 18 eyes (30.0%), which translates a continuous decrease of CRT over the months and years of ongoing treatment (Fig. 3). No correlation with the two investigated time parameters was seen in 12 (20.0%) eyes, and a significant influence of both parameters was found in 7 (11.7%) eyes.

**Table 2:** Demographic and clinical characteristics of eyes divided by outcome groups

	Number	Female proportion	Mean age (years) ± SD	Right eye proportion	Number of OCT measures available (mean ± SD)	Average of individual CRT means	Average of individual SD of CRT	Mean included time period (months ± SD)	Average of interval means since last injection (days ± SD)	Average of individual SD of intervals
All eyes	60	67%	78.8 ± 6.8	55%	15.6 ± 4.2	347 ± 100	35 ± 27	35.4 ± 8.8	30.3 ± 2.5	7.3 ± 2.5
Significantly positive CRT-interval correlation	25	68%	77.6 ± 6.4	60%	16.4 ± 4.7	325 ± 73	31 ± 23	35.7 ± 9.2	29.9 ± 2.5	8.0 ± 2.0
Significantly positive CRT-time correlation	12	67%	76.8 ± 5.6	33%	16.3 ± 4.1	335 ± 81	39 ± 25	30.8 ± 11.1	29.3 ± 2.3	6.8 ± 2.9
Significantly negative CRT-time correlation	18	67%	80.6 ± 6.3	61%	16.0 ± 5.0	364 ± 104	36 ± 35	38.6 ± 7.7	29.9 ± 2.9	7.7 ± 2.8

SD, standard deviation; CRT, central retinal thickness; interval, time between CRT measurement and preceding injection of anti-VEGF

The analysis of potential factors associated revealed the following: numerically, cases with intraretinal fluid showed more time-dependent change of CRT, however without reaching statistical significance—12 (53%) out of 23 eyes with intraretinal fluid showed an association of CRT with the time interval from the preceding injection, while the proportion in the absence of intraretinal fluid was 35% (*p* = 0.19). Those with presence of subretinal fluid seemed to show less frequently a correlation with the interval from last treatment (15 out of 42 eyes; 36%), again not significant (*p* = 0.15) compared with those without subretinal fluid (56%). No difference was seen for age, sex, and the anti-VEGF drug used. With regard to long-term evolution (time from baseline), the same observations were made: a non-significant, numerically higher proportion of eyes with cysts (15 out of 23 eyes; 65%; *p* = 0.10) showed time-dependent changes over the months and years than those without cysts (43%). Eyes with presence of subretinal fluid showed a slightly lower proportion of long-term changes (20 out of 42 eyes; 47%; *p* = 0.33) compared to those without subretinal fluid (61%). No difference was seen for age, sex, and the anti-VEGF drug used.

## Discussion

The present study about incomplete responders to anti-VEGF for nAMD, also referred to as refractory due to their intra- or subretinal fluid on OCT despite monthly retreatment, revealed that both time parameters, time interval from the preceding injection (42%) and time since first anti-VEGF injection (50%), influenced the CRT measurements. Their distribution showed some overlap, being relevant both simultaneously in some eyes.

CRT is a common outcome measure in many studies, including those about refractory nAMD. Nevertheless, the precise impact of time parameters has not been studied so far. We believe that influence of time parameters on CRT measurements may be important for study designs and interpretation, for two reasons: first, because nAMD is a dynamic disorder that evolves slowly over time, resulting in changes of CRT over time. In addition, anti-VEGF treatment has a relatively short half-life, and its effect on CRT may rapidly change when the critical concentration is reached. Thus, whenever CRT is studied as an outcome measure (or parameter), the influence of time on CRT should to be considered in the study design.

It is obvious to clinicians that time plays a role in increasing CRT with every nAMD recurrence following complete anti-VEGF response. The recurrences become more important within weeks to months requiring careful monitoring and an adequate retreatment regimen. However, when patients need monthly injections due to fluid present on each monthly visit, the impression may arise that this fluid is persistent, without any response to treatment. Such conclusion may be erroneous: the maximal fluid reduction is often achieved earlier than at 4 weeks [10]. Thus, there might be a group of patients presenting a transient responsiveness, with early recurrence and short-term CRT changes over days to weeks, related to the very short half-life of anti-VEGF agents. It might be interesting to follow these eyes with weekly OCTs. The widely used term “refractory” could therefore not be entirely precise, although this term is frequently used in order to describe the presence of fluid at monthly visits despite monthly treatment. However, with regard to monthly treatment being the maximal dosing for ranibizumab and aflibercept, the presence or reappearance of fluid may be considered to be a sign of incomplete responsiveness. As explained above, our finding of frequent correlation between CRT and time interval from last injection is biologically plausible. It was confirmed for 42% of cases. For those without such correlation, it is possible that they present truly persistent fluid non-responsive to anti-VEGF treatment, or that the collected data did not allow for discovering the correlation. The latter could be due to (1) a number of measurements too low, (2) an amount of fluid too small, and (3) a variability of time intervals too small for statistical significance (cf the standard deviation of the positive group was larger than for the entire cohort, therefore it was smaller for the negative group). Therefore, the real proportion of patients with short-term fluctuations of CRT after anti-VEGF injection may be higher than 42%. Whatever the precise proportion of short-term responsiveness is within the incomplete responders, there are at least two consequences to consider: First, studies about anti-VEGF refractoriness and using CRT as endpoint should benefit from considering the time since last injection. Second, without intermediate visit, it is not possible to determine whether the fluid seen at monthly visits is truly refractory to anti-VEGF. On the other hand, in case of truly persisting fluid in between injections, such fluid could be independent of VEGF. This population might still require anti-VEGF treatment to suppress excess fluid. However, the residual quantity of truly refractory fluid may be due to different pathways, including other exudative pathways, degenerative or inflammatory origin, polypoidal choroidal vascular changes, or AMD masquerading pathologies. Thus, an extensive imaging workup may be required. In case of polypoidal lesions which are best identified on indocyanine green angiography [11], an adjuvant treatment with photodynamic therapy might be a good option [12]. In addition, the inflammatory pathways have been shown to be involved in nAMD [13–16]. Although steroids are not the cornerstone in treatment of nAMD, their use as adjuvant therapy has been reported to be of interest [15].

The long-term effect of time from the monthly treatment is going on was also significantly associated with CRT in a high number of nAMD cases (50%) with incomplete response to anti-VEGF. Of these, in more than half, CRT decreased over time while less than half showed a slow CRT increase despite ongoing monthly treatment with anti-VEGF. This demonstrates that despite the refractoriness during certain periods, there might be change in CRT even without any change in treatment strategy. There are several implications of this finding: First, so-called refractory nAMD could improve over time with the same treatment. This might reflect slowly decreasing exudative activity of the underlying disorder. Thus, any study on the evolution of refractory nAMD over time would benefit from a control group. On the other hand, in cases of slow increase in CRT, the increasing exudative activity might indicate a developing drug tolerance that would potentially benefit from switching drugs [17]. The MARINA trial reported a loss of activity following multiple intravitreal injections along with the development of systemic antibodies in a subset of patients receiving ranibizumab [18]. Switching from one anti-VEGF to another, and sometimes back again to the previous one [19], has been reported to be of interest. However, drug tolerance is not the only possible explanation for slowly increasing CRT. The disorder may show changes in its exudative activity due to increased VEGF production or from other exudative pathways being upregulated over time.

Thus, it might be relevant not only to evaluate the patient’s response in comparison with the previous visit but also to evaluate the longer-term evolution. Distinguishing slow improvement from slow deterioration might allow differentiating between good prognosis and a situation requiring workup with possibly a treatment change, whether this is anti-VEGF switch or adjuvant treatment.

The analysis of potential factors did not reveal any clear associations. However, it was interesting to observe that the residual fluid of intraretinal location showed numerically more frequently a correlation with both time factors. We hypothesize that there might be a difference, for which our series was not large enough to be statistically significant. Recent studies revealed a different role of subretinal fluid versus intraretinal fluid, including poorer prognosis and more atrophy for intraretinal fluid [6, 20]. It has even been shown that subretinal fluid may be tolerated to a certain degree without loss of visual results [21].

Some limitations to this study are to be acknowledged. Besides the inherent weaknesses of any retrospective analysis, we admit that the real-life intervals of monthly treatment varied widely. This was however an indirect advantage in examining the dependency on time interval from the last injection. The use of the “observe and plan” regimen with its treatment series reduced the number of CRT measurements per time period but allowed the same number of CRT measurements to cover a large time period. In addition, the group was inhomogeneous for the anti-VEGF drug and the use of the OCT machine. However, within the individualized analysis, these two parameters were kept stable and are not expected to influence the results. However, a subgroup analysis was not possible, neither for the type of anti-VEGF drug nor for the type of refractory fluid in its quantity. The strength of the study includes the long time frame of refractoriness per patient available for analysis. In addition, all CRT measures were manually reviewed and corrected as needed, allowing for reliable and meaningful results.

In conclusion, time is a relevant parameter in the evaluation of the so-called refractory nAMD cases presenting incomplete response with intra- or subretinal fluid at monthly monitoring visits despite maximal monthly anti-VEGF treatment. Our study showed that refractory nAMD is not a homogeneous group of cases. There are short-term responders which can be distinguished from truly refractory cases using intermediate visits, for example, at 1 week after injection. The long-term trend for improvement or deterioration of CRT may be identified by observing a series of OCT images. Both aspects are promising parameters for adequate individual treatment adjustments, although precise recommendations are yet to be developed. In addition, studies exploring refractory nAMD would benefit from integration of the time parameters into the study design and analysis.

## Supplementary Information

ESM 1 (DOCX 36 kb)
