# Supplementary material for: Refractory neovascular age-related macular degeneration: time-dependent changes of central retinal thickness with anti-VEGF treatment
Source: Graefes Arch Clin Exp Ophthalmol. 2020 Nov 27;259(6):1477–86. doi: 10.1007/s00417-020-05000-3 (PMC8166689; doi:10.1007/s00417-020-05000-3)
Supplement: Supplementary file 1 — (DOCX 36 kb) [file 417_2020_5000_MOESM1_ESM.docx]

**Table 2**

Refractory neovascular age-related macular degeneration despite monthly retreatment with anti-VEGF, analyzed for central retinal thickness (CRT) measurements (manual correction of the segmentation) during refractoriness and its correlation with time variables from last injection and from baseline

| Patient study number | sexe | Age (years; at baseline) | eye | drug | OCT machine | range of refractoriness included (months from baseline) | OCT measures available (n) | mean CRT in µm (± SD) | Type of fluid with incomplete response | interval range since last injection (days) | mean interval since last injection (days (± SD)) | Correlation: CRT-interval (r) | Correlation: CRT-interval (*P* value) | correlation CRT - time point from baseline (r) | Correlation: CRT-time point from baseline (*P* value) | Multivariate analysis of time parameters if both *P*<0.2 |
| --- | --- | --- | --- | --- | --- | --- | --- | --- | --- | --- | --- | --- | --- | --- | --- | --- |
| 23 | M | 79 | L | RNZ | Cir | 3; 35 | 10 | 398 (± 53) | IRF | 27; 45 | 33 (± 6) | 0.85 | **0.002** | -0.04 | 0.900 | na |
| 18 | F | 77 | R | AFB | Sp | 7; 52 | 10 | 480 (± 18) | SRF | 23; 34 | 29 (± 3) | 0.76 | **0.010** | 0.22 | 0.540 | na |
| 17 | M | 88 | R | RNZ | Sp | 5; 35 | 10 | 335 (± 60) | SRF | 6; 49 | 31 (± 11) | 0.74 | **0.010** | 0.40 | 0.250 | na |
| 53 | M | 78 | R | RNZ | Sp | 8; 29 | 15 | 295 (± 28) | IRF, SRF | 7; 35 | 28 (± 6) | 0.70 | **0.004** | -0.33 | 0.240 | na |
| 51 | F | 65 | L | AFB | Sp | 40; 74 | 17 | 257 (± 15) | SRF | 15; 42 | 29 (± 9) | 0.67 | **0.003** | -0.33 | 0.190 | interval (*P* 0.002) |
| 5 | M | 77 | L | AFB | Sp | 3; 27 | 12 | 315 (± 12) | IRF | 22; 43 | 32 (± 6) | 0.67 | **0.017** | -0.19 | 0.560 | na |
| 15 | F | 73 | R | AFB | Sp | 3; 47 | 18 | 358 (± 19) | SRF | 26; 44 | 34 (± 6) | 0.66 | **0.004** | 0.43 | 0.090 | interval (*P* 0.006) |
| 39 | F | 83 | R | AFB | Sp | 49; 90 | 13 | 383 (± 52) | SRF | 11; 47 | 32 (± 9) | 0.65 | **0.016** | -0.42 | 0.150 | interval (*P* 0.01) |
| 45 | F | 64 | R | AFB | Sp | 22; 66 | 22 | 253 (± 53) | SRF | 10; 49 | 32 (± 8) | 0.62 | **0.002** | -0.04 | 0.870 | na |
| 20 | F | 82 | R | AFB | Sp | 3; 54 | 15 | 257 (± 6) | IRF | 18; 49 | 35 (± 8) | 0.61 | **0.016** | 0,28 | 0.310 | na |
| 33 | F | 89 | R | AFB | Sp | 16; 64 | 17 | 342 (± 27) | IRF | 8; 44 | 30 (± 10) | 0.58 | **0.010** | 0.21 | 0.420 | na |
| 38 | F | 83 | R | AFB | Sp | 39; 75 | 24 | 369 (± 40) | IRF | 7; 44 | 28 (± 9) | 0.57 | **0.004** | 0.22 | 0.290 | na |
| 13 | M | 81 | L | AFB | Sp | 3; 51 | 20 | 342 (± 39) | SRF | 7; 35 | 27 (± 9) | 0.57 | **0.008** | -0.27 | 0.250 | na |
| 54 | M | 82 | L | RNZ | Sp | 33; 55 | 14 | 376 (± 62) | SRF | 7; 38 | 28 (± 7) | 0.56 | **0.037** | 0.44 | 0.110 | interval (*P* 0.02) |
| 25 | M | 77 | R | RNZ | Cir | 3; 43 | 18 | 275 (± 13) | SRF | 24; 43 | 31 (± 4) | 0.55 | **0.020** | 0.41 | 0.090 | interval (*P* 0.03) |
| 37 | F | 74 | R | AFB | Sp | 64; 98 | 14 | 286 (± 17) | SRF | 8; 43 | 33 (± 10) | 0.54 | **0.049** | 0.01 | 0.980 | na |
| 8 | F | 85 | R | AFB | Sp | 5; 42 | 15 | 250 (± 7) | SRF | 7; 38 | 31 (± 7) | 0.54 | **0.036** | -0.37 | 0.190 | interval (*P* 0.02) |
| 46 | F | 85 | R | RNZ | Sp | 76; 103 | 19 | 281 (± 16) | IRF | 7; 45 | 27 (± 9 | 0.52 | **0.020** | -0.03 | 0.890 | na |
| 56 | M | 70 | L | RNZ | Sp | 40; 59 | 16 | 514 (± 32) | IRF, SRF | 7; 36 | 27 (± 8) | 0.55 | **0.026** | 0.48 | 0.061 | interval (*P* 0.01); **time (***P* **0.030)** |
| 22 | F | 81 | L | RNZ | Cir | 3; 35 | 23 | 221 (± 30) | IRF | 4; 42 | 29 (± 9) | 0.44 | **0.035** | 0.56 | **0.006** | interval (*P* 0.01); time (*P* 0.002) |
| 1 | F | 77 | L | AFB | Sp | 3; 27 | 10 | 238 (± 18) | SRF | 14; 42 | 30 (± 10) | 0.8 | **0.005** | -0.66 | **0.036** | interval (*P* 0.01) |
| 29 | F | 70 | R | AFB | Sp | 80; 122 | 24 | 285 (± 14) | IRF | 6; 35 | 27 (± 7) | 0.47 | **0.020** | -0.70 | **0.000** | interval (*P* 0.000); time (*P* 0.000) |
| 52 | F | 72 | L | AFB | Sp | 36; 80 | 25 | 357 (± 103) | IRF | 2; 47 | 27 (± 9) | 0.49 | **0.013** | -0.62 | **0.001** | time (*P* 0.006) |
| 18 | F | 77 | L | AFB | Sp | 3; 42 | 11 | 279 (± 8 | IRF | 7; 42 | 26 (± 11) | 0.75 | **0.010** | -0.77 | **0.008** | time (*P* 0.04) |
| 19 | F | 72 | R | AFB | Sp | 3; 47 | 18 | 385 (± 47) | SRF | 13; 48 | 31 (± 9) | 0.44 | 0.060 | 0.76 | **0.000** | **interval (*P* 0.030);** time (*P* 0.000) |
| 55 | F | 65 | R | RNZ | Sp | 3; 34 | 16 | 339 (± 18) | SRF | 7; 44 | 30 (±7) | 0.24 | 0.380 | 0.76 | **0.001** | na |
| 40 | F | 80 | L | RNZ | Cir | 8; 26 | 12 | 267 (± 22) | IRF | 26; 39 | 30 (± 4) | 0.31 | 0.320 | 0.72 | **0.008** | na |
| 14 | F | 77 | L | RNZ | Cir | 3; 35 | 22 | 261 (± 19) | SRF | 20; 36 | 30 (± 4) | 0.19 | 0.390 | 0.71 | **0.000** | na |
| 47 | M | 80 | L | AFB | Sp | 58; 78 | 9 | 412 (± 38) | SRF | 27; 32 | 29 (± 2) | -0.42 | 0.260 | 0.71 | **0.030** | na |
| 3 | M | 81 | L | AFB | Sp | 16; 33 | 15 | 257 (± 12) | IRF, SRF | 3; 46 | 24 (± 13) | 0.33 | 0.220 | 0.65 | **0.009** | na |
| 26 | M | 80 | L | RNZ | Sp | 81; 120 | 17 | 362 (± 69) | SRF | 7; 42 | 29 (± 7) | 0.28 | 0.270 | 0.62 | **0.007** | na |
| 4 | F | 72 | L | AFB | Sp | 3; 38 | 13 | 339 (± 63) | IRF | 16; 39 | 30 (± 6) | 0.29 | 0.390 | 0.6 | **0.032** | na |
| 6 | F | 82 | R | RNZ | Sp | 3; 34 | 14 | 308 (± 96) | SRF | 7; 42 | 29 (± 7) | -0.12 | 0.680 | 0.59 | **0.026** | na |
| 48 | F | 81 | R | AFB | Sp | 20; 73 | 20 | 357 (± 26) | SRF | 28; 44 | 34 (± 5) | 0.40 | 0.080 | 0.47 | **0.040** | n.s. |
| 35 | F | 87 | R | RNZ | Sp | 65; 98 | 15 | 527 (± 14) | SRF | 7; 35 | 27 (± 7) | 0.28 | 0.310 | -0.86 | **0.000** | na |
| 44 | F | 75 | R | AFB | Sp | 31; 67 | 14 | 471 (± 52) | IRF | 28; 44 | 32 (± 5) | 0.18 | 0.450 | -0.89 | **0.000** | na |
| 2 | F | 83 | R | AFB | Sp | 3; 29 | 10 | 327 (± 20) | IRF | 28; 35 | 30 (± 3) | -0.01 | 0.980 | -0.93 | **0.000** | na |
| 26 | M | 80 | R | RNZ | Sp | 81; 114 | 15 | 39 (± 131) | IRF | 7; 41 | 30 (± 8) | -0.12 | 0.680 | -0.96 | **0.000** | na |
| 34 | M | 78 | L | AFB | Sp | 14; 62 | 14 | 345 (± 28) | SRF | 28; 46 | 35 (± 7) | -0.51 | 0.060 | -0.81 | **0.000** | time (*P* 0.002) |
| 43 | F | 91 | R | AFB | Sp | 3; 35 | 12 | 544 (± 64) | SRF | 18; 36 | 29 (± 5) | 0.35 | 0.270 | -0.82 | **0.001** | na |
| 10 | F | 87 | R | AFB | Sp | 3; 47 | 12 | 248 (± 8) | SRF | 7; 49 | 33 (± 12) | 0.04 | 0.890 | -0.8 | **0.002** | na |
| 27 | M | 85 | R | AFB | Sp | 56; 89 | 14 | 307 (± 23) | SRF | 3; 49 | 27 (± 14) | -0.23 | 0.420 | -0.64 | **0.014** | na |
| 11 | M | 71 | R | RNZ | Sp | 3; 47 | 22 | 264 (± 28) | SRF | 7; 35 | 29 (± 6) | 0.28 | 0.200 | -0.5 | **0.017** | na |
| 9 | M | 79 | L | AFB | Sp | 6; 52 | 22 | 406 (± 24) | IRF | 1; 44 | 28 (± 9) | 0.36 | 0.100 | -0.46 | **0.030** | n.s. |
| 41 | F | 85 | L | AFB | Sp | 49; 96 | 20 | 402 (± 36) | IRF, SRF | 7; 42 | 29 (± 7) | 0.14 | 0.560 | -0.48 | **0.030** | na |
| 16 | F | 80 | R | AFB | Sp | 3; 54 | 20 | 295 (± 12) | IRF | 24; 49 | 32 (± 7) | 0.43 | 0.060 | -0.49 | **0.030** | time (*P* 0.004) |
| 36 | M | 90 | R | AFB | Sp | 70; 105 | 11 | 570 (± 64) | SRF | 28; 45 | 36 (± 5) | 0.09 | 0.790 | -0.64 | **0.030** | na |
| 21 | F | 84 | L | AFB | Sp | 3; 41 | 17 | 291 (± 9) | IRF | 28; 46 | 32 (± 6) | 0.01 | 0.970 | -0.52 | **0.033** | na |
| 49 | F | 73 | R | AFB | Sp | 19; 57 | 14 | 194 (± 35) | IRF | 6; 47 | 30 (± 9) | -0.13 | 0.670 | -0.5 | 0.070 | na |
| 49 | F | 73 | L | AFB | Sp | 3; 41 | 13 | 264 (± 12) | IRF | 21; 44 | 32 (± 8) | -0.18 | 0.560 | -0.49 | 0.090 | na |
| 30 | F | 74 | L | AFB | Sp | 41; 72 | 14 | 427 (± 28) | SRF | 21; 38 | 31 (± 4) | -0.51 | 0.060 | 0.45 | 0.110 | n.s. |
| 50 | M | 80 | L | RNZ | Sp | 16; 49 | 16 | 318 (± 59) | SRF | 2; 45 | 28 (± 11) | 0.47 | 0.060 | 0.4 | 0.130 | n.s. |
| 24 | F | 80 | R | RNZ | Cir | 3; 35 | 15 | 423 (± 85) | IRF | 26; 35 | 30 (± 3) | 0.47 | 0.080 | -0.39 | 0.170 | n.s. |
| 12 | F | 87 | R | AFB | Sp | 3; 49 | 17 | 278 (± 5) | SRF | 28; 42 | 32 (± 4) | 0.44 | 0.080 | -0.28 | 0.280 | na |
| 28 | M | 78 | R | AFB | Sp | 29; 65 | 11 | 296 (± 37) | SRF | 28; 47 | 34 (± 7) | -0.18 | 0.590 | -0.35 | 0.280 | na |
| 7 | F | 85 | R | RNZ | Sp | 33; 58 | 9 | 355 (± 14) | IRF, SRF | 26; 47 | 32 (± 6) | 0.46 | 0.210 | -41 | 0.280 | na |
| 42 | M | 84 | L | RNZ | Cir | 15; 45 | 12 | 248 (± 9) | SRF | 27; 47 | 33 (± 6) | 0.13 | 0.690 | 0.24 | 0.450 | na |
| 31 | F | 70 | L | AFB | Sp | 33; 71 | 16 | 461 (± 80) | IRF | 10; 42 | 32 (± 8) | -0.32 | 0.230 | 0.1 | 0.700 | na |
| 43 | F | 91 | L | AFB | Sp | 32; 64 | 11 | 750 (± 24) | SRF | 18; 36 | 29 (± 5) | -0.41 | 0.210 | 0.02 | 0.960 | na |
| 32 | F | 61 | L | AFB | Sp | 29; 64 | 21 | 386 (± 38) | SRF | 7; 48 | 31 (± 10) | 0.03 | 0.890 | 0 | 0.980 | na |

Legend

L, left eye; R, right eye; RNZ, Ranibizumab; AFB, Aflibercept; Cir, Cirrus Zeiss; Sp, Spectralis Heidelberg; baseline, date of anti-VEGF treatment initiation; CRT, central retinal thickness; IRF, intraretinal fluid; SRF, subretinal fluid; SD, standard deviation; r, Pearson correlation coefficient; na, not applicable, n.s., not significant
